# “Being an informal caregiver – strengthening resources”: mixed methods evaluation of a psychoeducational intervention supporting informal caregivers in palliative care

**DOI:** 10.1186/s12904-024-01428-0

**Published:** 2024-04-11

**Authors:** Tabea Theißen, Anneke Ullrich, Karin Oechsle, Julia Wikert, Carsten Bokemeyer, Aneta Schieferdecker

**Affiliations:** 1 Palliative Care Unit, Department of Oncology, Haematology and BMT, University Medical Centre Hamburg-Eppendorf, Martinistr. 52, 20246 Hamburg, Germany; 2 Department of Palliative Medicine, LMU University Hospital, Munich, Germany

**Keywords:** Informal caregivers, Palliative care, Psychoeducational intervention, Pilot study, Empowerment

## Abstract

**Background:**

Informal caregivers are key support for patients with progressive incurable diseases. However, their own needs often remain unmet. Therefore, we developed, manualised and implemented the intervention “Being an informal caregiver – strengthening resources” aiming to support and empower informal caregivers by addressing relevant information-related, physical, psychological and social needs.

**Methods:**

In this pilot study, we evaluated the acceptance and experiences with this psychoeducational intervention. The study was conducted over two years (2019-2021). Informal caregivers were recruited from the University Medical Centre Hamburg-Eppendorf and the metropolitan region of Hamburg, Germany. The intervention was aimed at adult persons who identified themselves as an informal caregiver to an adult patient with a progressive incurable cancer and non-cancer disease. For the evaluation we used a mixed methods approach, combining a longitudinal questionnaire survey (pre-intervention, after each module, 3-months follow-up) and semi-structured interviews post-intervention. Quantitative data were analysed using descriptive statistics and a paired *t*-Test, interviews were analysed based on the qualitative content analysis according to Mayring. Results were triangulated using a convergent triangulation design.

**Results:**

Of 31 informal caregivers who received the intervention, 25 returned the follow-up questionnaire and 20 informal caregivers were interviewed. Triangulated results showed a high satisfaction with the implementation of the intervention. Of a broad range of subjective benefits, gaining knowledge, self-awareness and self-efficacy were most apparent. Informal caregivers reported improved preparedness, awareness of own needs as well as confidence regarding handling own emotions and interacting with the ill person. However, implementing the learned skills into daily life can be challenging due to internal and external factors. Motivations and challenges for participating as well as potential for improvement were identified.

**Conclusions:**

This pilot study showed an overall positive evaluation and several subjective benefits of the psychoeducational intervention “Being an informal caregiver – strengthening resources”. Further research is needed to measure the efficacy of this intervention on informal caregivers’ outcomes. Therefore, a multicentre randomized prospective study is planned.

**Supplementary Information:**

The online version contains supplementary material available at 10.1186/s12904-024-01428-0.

## Introduction

Informal caregivers (ICs) are key support for patients with progressive incurable cancer and non-cancer diseases [1–3]. At the same time, ICs are affected by the patients' disease with own burden and needs: Studies demonstrate various psychological, social, physical and economic burden as well as health system challenges of these ICs [1–10]. ICs psychosocial burden may even exceed that of the patients [4, 11, 12] and increases with the patients’ disease progression and nearing death [12–16]. This is reflected in a high prevalence of anxiety (32% - 47%) and depression (20% - 41%) in ICs [5, 6, 17–19]. Unmet needs are associated with significantly higher levels of ICs psychological and overall caregiver burden [20–22]. Most common unmet needs are informational needs, especially those related to the patients’ disease and its treatment as well as care issues [23, 24]. Furthermore, ICs physical, psychological, financial, spiritual and social unmet needs are well described [23]. Being an IC means enormous responsibility and many ICs feel unprepared at the practical and the emotional levels of caring for a patient with progressive incurable disease [25]. Empirical evidence shows that feeling prepared is associated with decreased IC burden [25–27]. Therefore, it is paramount to improve ICs knowledge and competence to facilitate preparedness, support role recognition and increase confidence in ICs [28].

Increasingly, international studies assess interventions for ICs aiming at addressing their burden and needs during palliative care [1, 29]. Previous interventions were structured in different ways (psychoeducational interventions, behavioural or skills trainings and palliative care team interventions), delivered at group or individual level, and focused different target groups (ICs, IC-patient dyads or patients plus their families) [1]. Some studies have already shown that interventions can have positive effects on self-efficacy and preparedness [30–33] as well as on psychological burden [29, 34, 35] in ICs. However, many studies are pilot or feasibility studies and results are inconclusive [1, 29]. Meta-analyses show mixed results regarding ICs psychological burden, including distress, anxiety and depression, and quality of life [36–38]. Focus on heterogeneous populations, heterogeneous interventions contents and implementations as well as numerous or unspecified primary end points make it difficult to draw conclusions or to recommend one particular intervention [1].

In Germany, evaluated interventions for ICs in palliative care are limited. The 22-hours existential behavioural therapy of Fegg et al. demonstrated significant effects on anxiety, depression and quality of life [39]. However, the 2-hours short-term therapy showed no significant impact on ICs outcome [40]. The educational initiative “Last Aid Course” (LAC) by Georg Bollig aims to teach the public about the fundamentals of palliative care. It has been implemented in 20 different countries, including Germany [41]. LAC can lead to the promotion of death literacy in communities and is feasible in a web-based format [42, 43]. To date, available data on the effects of LAC on ICs are preliminary, especially in Germany [44, 45].

Because of this gap in evaluated interventions for ICs of patients with progressive incurable cancer and non-cancer diseases in Germany, we developed, manualised and implemented the psychoeducational intervention “Being an informal caregiver – strengthening resources” and conducted a mixed methods pilot study. The aim of this study was to evaluate ICs acceptance of and experiences with the intervention, including participation, motivation and for participating, satisfaction with the implementation, subjective benefits and potential for improvement.

## Methods

### Intervention

#### Design of the intervention

The aim of the newly developed psychoeducational intervention “Being an informal caregiver – strengthening resources” was to support and empower ICs of patients with incurable progressive diseases by increasing their knowledge, self-awareness and self-efficacy. Self-awareness is the ability to focus on oneself and how the own actions, thoughts or emotions align with internal standards [46] and self-efficacy is the belief in one’s ability to confidently deal with a specific situation [47]. We wanted to develop an intervention that would be as efficient as possible, but also practical in many places. At the same time, it has to be appealing and helpful for many and ICs should be able, to share their experiences with others in a group setting.

The intervention setting was an ongoing group program, including six multidisciplinary modules (90 min. duration each; table 1). Each week on the same day (Monday 5pm), one of the six modules was offered. ICs could join the program at any time and in any order as well as individually decide which modules to attend. The contents of the modules covered the physical, psychological, social and spiritual dimensions of palliative care. While most of them are directly visible in the topics of the modules (e.g. physical in module 1 and 5, table 1), others can be found all over the modules. For instance, spirituality: seeing and validating the ICs as individuals, encouraging and supporting them to approach emotions such as grief, loss and fears as well as finding and using resources. The modules were offered by palliative care professionals with expertise in different fields (physician, nurse, grief counsellor, social worker, physiotherapist, psychologist). To ensure the intervention fidelity, weekly implementation of the modules was accompanied and moderated by one member of the research team (psychologist).

**Table 1:** Modules of the intervention “Being an informal caregiver – strengthening resources”

**Module No.**	**Topic**	**Module content (examples)**
1	Hands-on care: tips and strategies for **providing care at home** (nurse)	- Caring for a patient with progressive incurable diseases
		- Which symptoms to expect?
		- Handling different symptoms (e.g. loss of appetite, dry mouth)
		- Where to find further practical tips?
2	Getting prepared: Information about **social and legal issues** (social worker)	- Different types of care (e.g. outpatient palliative care, hospice)
		- Patient decrees and health care proxy
		- What financial assistance can I get and how?
		- Where to get further information?
3	Questions, uncertainties and concerns about **grief and loss** (grief counsellor)	- Grief and bereavement
		- What can I do for myself?
		- How can I support significant others?
4	Strategies to cope with own **needs and emotions** (art therapist/psychologist)	- Burden and needs of ICs
		- Fear, anxiety, sadness, grief, worries
		- Coping: double awareness
		- Practical exercises for finding and using own resources
5	Strategies for handling **changes in the disease progression** (physician)	- When does palliative care start?
		- (Non-)pharmacological therapies for managing different symptoms (e.g. pain, fatigue)
		- Handling medications
6	Practical exercises for **self-care and own physical well-being** (physiotherapist)	- Importance of physical activity (caring for oneself is caring for others)
		- Practical tips for daily life
		- Movement and relaxation exercises

The intervention was offered from November 2019 until November 2021. Initially, it was conducted in a face-to-face format. Due to the COVID-19 pandemic, it was paused from end of March until June 2020. During that time, the intervention and evaluation components were adjusted into a web-based format. From July 2020, the intervention was offered in a web-based format as videoconference

#### Development of the intervention

The intervention was based on the curriculum for developing training programs in palliative care (KoMPaC) by the German Association for Palliative Medicine [48]. Additionally, experts of the study team reviewed IC-directed interventions in the context of palliative care. Palliative care experts were involved in the planning process to increase practical relevance of the intervention. In the end, six standardised evidence-based modules were developed. For each module, learning objectives, contents, and materials, including presentations and handouts, were developed and manualised. Handouts for ICs included further recommendations, like information about support services and recommended reading. The materials were iteratively revised through feedback from palliative care experts during the development plus throughout the study, as we asked participating ICs and palliative care experts for feedback, including those who delivered the intervention. There were regular discussions and exchanges between the palliative care experts and the moderator. We used the TIDieR (Template for Intervention Description and Replication) Checklist (Suppl. File 1) to report on the intervention [49].

### Study design

**Fig. 1:**
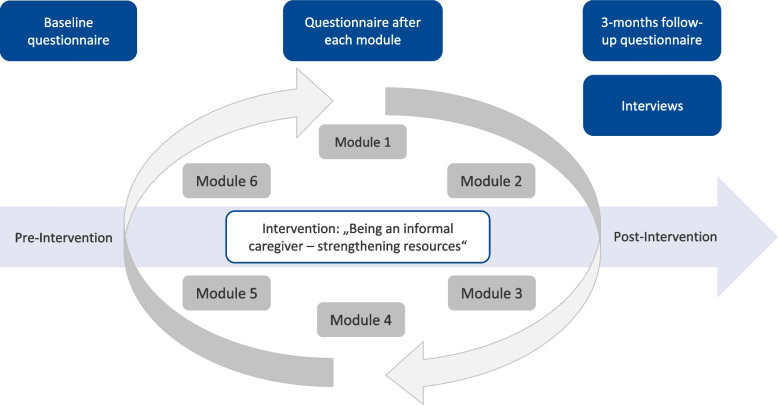
Study design

This prospective interventional mixed methods study was conducted between November 2019 and November 2021. It combines a quantitative study part, including a longitudinal questionnaire survey at baseline (pre-intervention), after each module and at follow-up three months after baseline as well as a qualitative study part with semi-structured interviews post- intervention. As shown in Fig. 1, the six modules were offered as an ongoing program, one module per week. After six weeks, the program started from the beginning, as illustrated by the two arrows forming a cycle. ICs could join the intervention at any time and in any order.

The mixed methods design approach was applied to triangulate the study, as mixed methods designs have been increasingly used in palliative care research to enrich findings [50]. Quantifying the ICs experience solely by questionnaires might be weak in understanding reasons behind their experiences with the intervention. Therefore, qualitative research was used for a deeper insight into the ICs subjective experience. To combine the approaches, we used a convergent triangulation method. The convergent triangulation design is a mixed methods design in which quantitative and qualitative data are collected and analysed separately and then results from both databases are compared and merged for interpretation [51].

The ethics committee of the Medical Association in Hamburg, Germany approved the study (June 04, 2019; Reference: PV7009).

### Participants

The intervention was addressed to ICs of patients with a progressive incurable disease. All individuals who identified themselves as a primary IC could participate. ICs are characterised as people providing unpaid care to someone with whom they have a personal relationship with (family, friend or another close person) [52]. We did not specify further criteria of informal caregiving (like duration, kind or effort of taking care). ICs were recruited from the University Medical Centre Hamburg-Eppendorf and the metropolitan region of Hamburg, Germany, via information material (flyers and posters). We used a wide approach to reach as many ICs as possible (e.g. medical practices, pharmacies, supermarkets). Interested ICs were asked to contact the study team, who informed about the study and checked eligibility. Inclusion criteria were: being an adult primary IC (≥18 years) to an adult patient with a progressive incurable disease and cognitive capacity for giving fully informed consent and completing the questionnaires. Written informed consent was obtained from all participating ICs. Within the informed consent, ICs were asked, if they would agree to be contacted for the interview later on. All ICs, who participated in an interview, gave informed consent.

### Data collection

#### Procedures of data collection

ICs, who registered for the intervention, received the baseline questionnaire (pre-intervention) and the 3-months follow-up questionnaire by mail. In addition, they received a questionnaire directly after each module. In the face-to-face format, questionnaires were handed out and filled in on-site. After web-based modules, ICs received a link for the online survey.

Participants for the interviews were selected using a purposive sampling (criteria: number of participated modules: at least 5 vs. less, format of participation: web-based vs. face-to-face). Two female researchers (AS: physician, TT: psychologist), who were not known by the participants, interviewed the ICs via telephone. Interviews were planned to last 45 min., were audio-recorded with permission and transcribed verbatim. Besides, field notes were made during the interviews.

#### Measurements

##### Sociodemographic and medical variables

ICs informed about their age, gender, current occupation, school education, relationship to the patient, as well as the patient’s disease, place of residence and care level.

##### Process data

Data regarding the intervention format, module and date of participation were prospectively documented.

##### Satisfaction with the implementation of the intervention

The *Health Education Impact Questionnaire program scale* (heiQ-program) was used to assess the quality of the intervention implementation [53]. It includes 9 items, each rated on a 6-point Likert scale (0 “strongly disagree” to 5 “strongly agree”). The heiQ-program was reported on a single item level. Reliability and validity have been supported for the German Version [54]. The internal consistency of the heiQ-program in this study was satisfying (Cronbach’s alpha .89; original heiQ .08 or higher). Using the heiQ format, we developed two additional items, asking ICs whether their expectations had been met and whether the subject matter provided was clear and easy to understand. The two additional items were the result of testing the questionnaire with two ICs during the development phase of the intervention in order to check its completeness and comprehensibility. The heiQ-program plus the two additional items were used at follow-up as well as after each module.

##### Subjective benefits

Due to the lack of suitable instruments, we used eight self-developed items to investigate ICs perceived benefits of participating. Items were rated in accordance with the heiQ-program (0 “strongly disagree” to 5 “strongly agree”). For example, ICs indicated whether they have more answers to important questions, know where to find support as an IC and feel more confident. These items were used at follow-up as well as after each module. Additionally, ICs rated how helpful the intervention was (scale: 1 “not helpful at all” to 10 “extremely helpful”) at follow-up.

##### Caregiver burden

ICs subjective burden of care was evaluated using the validated German short version of the *Burden Scale for Family Caregivers* (BSFC-s) [55]. The 10 items are rated on a 4-point Likert scale (0 “strongly disagree” to 3 “strongly agree”). The score ranges from 0 to 30, with higher scores indicating greater burden (low: 0-5, medium: 6-14, high 15-30). The BSFC-s was used at baseline and follow-up. Cronbach’s alpha in this study was satisfying (baseline .90; follow-up .86; original BSFC-s .92 [55]).

##### Interview guide

A semi-structured interview guide (Supplement File 2) was used for deeper insight into the ICs experience with the intervention. Topics are displayed in Table 2.. The guide was developed based on current literature as well as practical clinical knowledge, following the approach by Helfferich [56]. During implementation, no need for further modification of the guide emerged.

**Table 2.** Topics of the semi-structured interview guide

1. Introducing the topic and conduct of the interview
2. Questions to generate/validate participant information (e.g. number of modules attended)
3. Exploration of motivations and expectations before the intervention
4. Perceived positive and negative aspects of the intervention
5. Challenges and difficulties of participating in the intervention
6. Supporting and hindering factors for practical implementation into daily life
7. Exploration of further topics and wishes regarding the intervention
8. Closing of the interview

### Data analysis

Descriptive statistics were calculated including the frequencies, percentages, means (*M*) and standard deviations (*SD*). The heiQ-program plus the two additional items were analysed on a single item level with ratings of 4 (“agree”) and 5 (“strongly agree”) being considered as satisfaction with the intervention. IC burden was analysed using the mean total score of the BSFC-s. The authors of the BSFC-s used EM algorithm for imputing missing values [57]. Due to our small sample size, we refrained from that and decided on replacing the mean score on an individual level [58]. Therefore, scores for respondents with one or two missing items were calculated by imputing the mean score for the missing items based on items completed by that individual. A paired *t*-Test was conducted to investigate changes in ICs burden before and after the intervention. All conditions required to conduct a t-test were met [59]. All statistical analyses were computed using IBM SPSS Statistics version 27.0 [60] and a significance level of α < 0.05.

Interviews were iteratively analysed based on the qualitative content analysis according to Mayring [61], using the software program MAXQDA (2020). The analysis took place using inductive coding techniques. A preliminary coding template was developed based on the line by line coding of a subset of transcripts (50% of the data material) by one researcher (AS). Categories and subcategories were regularly discussed in a series of meetings by the multi-professional research team. As no new (sub-) categories emerged after the tenth interview, all interviews were coded using the final coding template in accordance to Mayring [61]. To ensure intra- and interrater reliability, two researchers (TT, AS) each coded the data of five transcripts independently. The comparison showed high agreement between the two researchers; differences were resolved in discussions within the research team (TT, AS, AU).

#### Convergent triangulation design

In accordance to Creswell and Clark [51], quantitative and qualitative data were collected and analysed parallel. Afterwards the two sets of results were merged and jointly interpreted in a narrative discussion. Throughout a series of meetings (TT, AS, AU, KO), data was compared and contrasted. We looked for common concepts across the results and compared quantitative and qualitative results side-by-side for each concept to identify in what ways they confirm, disconfirm or expand each other. We used the guidelines for Good Reporting of A Mixed Methods Study (GRAMMS) Checklist [62] (Supplement File 3).

## Results

### Characteristics of participants and participation

Of 36 ICs registered for the intervention, five dropped out before baseline. In one case, the patient died before the IC started the intervention; in a further four cases, the reasons for dropout remain unknown, as the ICs could not be contacted despite several attempts. The remaining 31 ICs completed the baseline questionnaire and were included in the intervention, with 25 ICs returning the follow-up questionnaire. Therefore, six ICs were lost to follow-up for reasons unknown. Afterwards, 20 of the 31 ICs were interviewed (duration in min.: *M*=31.8, *SD*=12.1, 15-62). The mean age of participating ICs (*N*=31) was 51.4 years and 90% (*n*=28) of ICs were female. In most cases, the person they cared for was living at home (*n*=25, 81%), was their spouse (*n*=20, 65%) and had an underlying cancer disease (n=21, 68%; Table 3).

**Table 3:** ICs and patient-related characteristics at baseline (*N*=31)

*Informal caregivers characteristics*		
Age, *M* (*SD*); Range in years		51.4 (12.4); 27-75
Age in Groups, *n* (%)	27-39	7 (22.6)
	40-49	4 (12.9)
	50-59	12 (38.7)
	60-69	7 (22.6)
	70-75	1 (3.2)
Gender identity, *n* (%)	Female	28 (90.3)
	Male	3 (9.7)
Current occupation, *n* (%)	Not working	7 (22.6)
	Working full time	14 (45.2)
	Working part-time	7 (22.6)
	Missing	3 (9.7)
School education^a^,* n* (%)	Low (≤ 9 years)	1 (3.2)
	Intermediate (10 years)	9 (29.0)
	High (12-13 years)	21 (67.7)
Relation to patient, n (%)	Spouse	20 (64.5)
	Child	5 (16.1)
	Other^b^	5 (16.1)
	Missing	1 (3.2)
*Patient-related characteristics*		
Patient’s disease, *n* (%)	Cancer	21 (67.7)
	Non-cancer^c^	5 (16.1)
	Cancer and non-cancer^c^	4 (12.9)
	Missing	1 (3.2)
Patient’s place of residence, *n* (%)	At home	25 (80.6)
	Nursing home	3 (9.7)
	Other	2 (6.5)
	Missing	1 (3.2)
Patient’s need for physical care,* n* (%)	No care needed	12 (38.7)
	Informal caregiver him/herself	10 (32.3)
	Involvement of others (nursing service and/or social environment)	5 (16.1)
	Missing	4 (12.9)

*Abbreviations*: *M* Mean, *SD* Standard deviation

^a^low: secondary general school leaving certificate or less, intermediate: intermediate school-leaving certificate, high: university entrance qualification

^b^Relation to patient “other”: parent, sibling, grandchild

^c^Patient’s disease “non-cancer”: coronary heart disease, heart attack, heart transplant, progressive multifocal leukoencephalopathy, multimorbid

Table 4 summarizes the characteristics of participation. Of 31 ICs, 14 (45%) completed the intervention (=at least 5/6 modules). However, this is more a description of the use of the intervention than a quality indicator, as ICs were able to select modules according to their needs. The average number of attended modules was 3.6 out of 6 (*SD*=2.0, range 1-6). Due to the pandemic, 19 (61%) of the ICs participated web-based. The average duration of participation was 15.5 weeks (*SD*=4.5, range 1-27). In relative terms, module 2 (*Getting prepared: Information about social and legal issues*) and module 5 (*Strategies for handling changes in the disease progression*) were attended most commonly, by 21 ICs (68%) each.

**Table 4:** Characteristics of participation (*N*=31)

*Characteristics of participation*		
Duration of participating, *M* (*SD*); Range in weeks		15.5 (4.5); 1-27
Number of attended modules,* M* (*SD*)		3.6 (2.0)
Number of participants completing the intervention ^a^, *n* (%)		14 (45.2)
Number of participants not completing the intervention ^b^, *n* (%)		17 (54.8)
Format of the intervention, *n* (%)	Web-based	19 (61.3)
	Face-to-face	12 (38.7)
Number of participants per module in total, *n* (%) attended	Module 1	17 (54.8)
	Module 2	21 (67.7)
	Module 3	18 (58.1)
	Module 4	19 (61.3)
	Module 5	21 (67.7)
	Module 6	17 (54.8)

*Abbreviations*: *M* Mean, *SD* Standard deviation

^a^completing the intervention: participated in at least 5 out of 6 modules

^b^not completing the intervention: less than 5 modules

#### Triangulated results of the evaluation

**Fig. 2:**
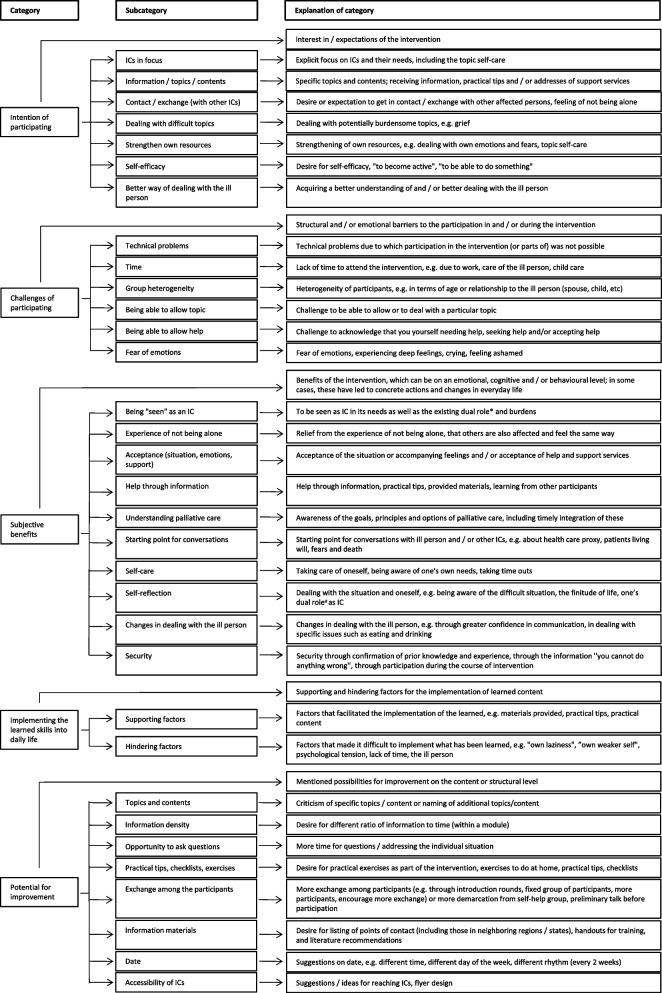
Category system of the qualitative evaluation. Abbreviations: ICs, informal caregivers; ^a^ dual role = being a person who supports and cares for the ill person at the same time as being a person with own needs

In the following, triangulated results are reported. The triangulation showed that in some parts qualitative analysis gave results in greater detail, in other parts quantitative analysis was more informative and sometimes both parts were complementary. Qualitative findings are displayed in Fig. 2. In supplement file 4, the detailed category system is provided and explanations and illustrative quotes for each subcategory have been added. Quantitative data are summarized in Tables 5, 6 and 7, data on module level in Supplement File 5. Tables and figures are shown where the corresponding results are reported.

**Table 5:** Satisfaction with the implementation of the intervention

**Items**	***M* (*SD*)**	**Range**^**a**^	**Agree**^**b**^ ***n/N* (%)**
**HeiQ-program**			
1. I will tell people that the intervention is very worthwhile.	4.6 (0.8)	2-5	23/25 (92.0)
2. The Intervention has helped me set reasonable and achievable goals.	3.7 (0.7)	2-5	16/25 (64.0)
3. I trust the information and advice given in the intervention.	4.7 (0.5)	4-5	25/25 (100.0)
4. The intervention was very well organized.	4.4 (0.7)	3-5	22/25 (88.0)
5. Taking part in the intervention was worth my time and effort.	4.4 (0.7)	3-5	22/25 (88.0)
6. Difficult topics and discussions were handled well.	4.3 (0.6)	3-5	23/25 (92.0)
7. The intervention content was very relevant to me and my situation.	4.1 (0.8)	3-5	19/25 (76.0)
8. Everyone had the chance to speak if they wanted to.	4.5 (0.8)	2-5	23/25 (92.0)
9. The group worked very well together.	4.0 (0.9)	2-5	19/25 (76.0)
**Additional items**			
10. My expectations of the intervention were met.^c^	3.8 (0.8)	2-5	18/24 (75.0)
11. The subject matter provided was clear and easy to understand.^c^	4.6 (0.5)	4-5	25/25 (100.0)

*Abbreviations*: *M* Mean, *SD* Standard deviation

^a^range on a Likert response scale from (0 *strongly disagree -* 5 *strongly agree*)

^b^versus (*strongly) disagree/disagree somewhat/agree somewhat*

^c^Study specific items not included in the original heiQ-program

**Table 6:** ICs benefits of participating

		***M* (*SD*)**	**Range**^**a**^	**Agree**^**b**^
**Topic**	*Comparing my current situation to my situation before the intervention, I now...*			***n/N* (%)**
**Gaining knowledge**	know better where to find help and support as a ICs	3.9 (0.8)	2-5	18/24 (75.0)
	have more answers to important questions	3.9 (0.9)	2-5	17/24 (70.8)
**Self-awareness and -efficacy**	am more aware of my own needs as a ICs	3.7 (0.9)	2-5	15/23 (65.2)
	feel more confident handling feelings regarding this topics	3.5 (0.9)	2-5	15/24 (62.6)
	feel more confident interacting with the ill person	3.4 (1.1)	1-5	11/23 (47.8)
	am better at estimating, what I am capable of	3.5 (0.8)	2-5	11/24 (45.8)
	know my own abilities better now	3.2 (1.1)	0-5	9/24 (37.5)
**Potential burden**	do NOT feel more anxious, worried or depressed	4.0 (0.9)	1-5	19/23 (82.6)

*Abbreviations*: *M* Mean, *SD* Standard deviation, *ICs* Informal caregivers

^a^range on a 6-point response scale from (0 *strongly disagree -* 5 *strongly agree*)

^b^versus (*strongly) disagree/disagree somewhat/agree somewhat*

**Table 7:** Supporting factors for practical implementation of the learned skills into daily life

*What helped you implementing what you have learned in the intervention?* *Yes (multiple answers possible)*	***n/N* (%)**
Myself/my own motivation	23/25 (92.0)
Other affected persons	12/25 (48.0)
Information from TV, books, brochure, etc.	11/24 (45.8)
The ill person	10/24 (41.7)
Physician or other healthcare professionals	10/24 (41.7)
Friends	10/25 (40.0)
Additional support services	8/23 (34.8)
Family, Children, Grandchildren	6/25 (24.0)

### Intention of participating

In the qualitative data, ICs reported expectations and motivations for participating in the intervention (Fig. 2). One motivational factor was the explicit focus of the intervention on ICs, as their role as ICs, associated burden and needs are often overlooked:

*“Everyone naturally cares about the ill person and asks about him, how he is going, but no one asks about how I am.”* (IC 04)

ICs also expected to receive information about specific topics, e.g. care issues, legal aspects and support services. They believed that an increased knowledge on these topics could help them being more prepared and confident. For example, one IC wanted to acquire information about what she might face in future:

*„Information, gaining knowledge (...) I just wanted to know: what to expect and who to ask if I don't know what to do.”* (IC 24)

Likewise, ICs expected to learn how to deal with potentially burdensome topics (e.g. grief), own emotions and fears, as well as finding a better way of understanding and dealing with the ill person. They hoped to strengthen their resources by improving self-care and self-efficacy. Furthermore, ICs participated in the intervention to get in contact with others concerned, to share experiences and feel less isolated.

### Satisfaction with the implementation of the intervention

The evaluation of the heiQ-program showed medium to high satisfaction with the implementation of the intervention (Table 5). Overall, 8 of the 9 original heiQ-program items were rated as “agree” by at least 76% of ICs. Satisfaction was particularly visible for module 1 (*Hands-on care: tips and strategies for providing care at home*), for detailed data see Supplement file 5. At follow-up, all ICs (*n*=25, 100%) reported that they have trusted the given information and felt that the subject matter provided was clear and easy to understand. Interview data refined that ICs trusted the given information as they had trust in the institution delivering the intervention:

*„A program at an institution that I consider trustworthy, namely a university hospital (...), and I have the opportunity to inform myself about the broad spectrum of issues, that's a great chance for me.”* (IC 24)

On average, ICs rated the whole intervention as very helpful (*M*=8.2, *SD*=1.6, range: 3-10 on a scale of 1 “not helpful at all” – 10 “extremely helpful”; data not shown). Overall, 92% (*n*=23) would recommend the intervention to other ICs.

ICs described feeling encouraged by the palliative care experts delivering the intervention, as besides receiving new information, they felt validated in their prior knowledge and experiences. They also reported gaining confidence over the course of the intervention, through the way the course was implemented and the way the experts act.

“*At the beginning I was skeptical, maybe a little bit afraid, because of the emotional issues, but in the course it was okay, I felt really well taken care of.”* (IC11)

### Subjective benefits of the intervention

#### Changes on cognitive, emotional and behavioural level

Qualitative data revealed that subjective benefits of the intervention can be described on three levels: cognitive, emotional and behavioural.

On the cognitive level, beneficial changes due to the intervention included increase in knowledge, self-reflection and self-awareness:

*„Due to the intervention, I realised that I should take care of myself more often.“ (IC 04)*

On the emotional level, beneficial changes included a sense of encouragement (e.g. to start therapy), strength, gratitude, feeling less guilty (e.g. when doing self-care activities), and better acceptance of one’s own emotionality:

*„(…) to gain the feeling that it is okay to feel a certain way and also to accept oneself with one's own fears and worries.“* (IC 33)

Further, the intervention had led to goal directed behaviour and concrete actions in ICs everyday life. For instance, including more self-care into daily life, significant changes in dealing with the ill person and use of support services (e.g. psychotherapist, palliative care specialist):

*„We immediately looked for a local palliative care physician, here where we live (...) we go there every four weeks.“* (IC 24)

#### Topics of subjective benefits

Several perceived benefits of participating in the intervention could be identified in quantitative (Table 6) and qualitative data (Fig. 2). Quantitative results appeared similarly across all modules (for detailed data see Supplement File 6).

##### Gaining knowledge

Quantitative analysis demonstrated a subjective benefit of the intervention regarding the ICs knowing more about support services for ICs (*n*=18, 75%) and having more answers to important questions (*n*=17, 71%; Table 6). Qualitative data support these findings, as ICs reported that the intervention helped them through information about topics such as legal aspects, care issues, disease progression and medication, and support services. Thus, ICs felt better prepared and relieved:

*„I learned (...) who I can contact and also how it works when he dies, who will take care of him and that the funeral home will do a lot. That was a real benefit for me.“* (IC 05)

Having more knowledge was also described as a good starting point for discussions with the ill person as well as with others concerned. Realising the principles and goals of palliative care led to relief and more acceptance of help, as one participant reported:

*“I realized (…) that palliative care does not just start when the end of life is very near, but even before that (...) that was a very important realisation for me.“* (IC 28)

##### Self-awareness and -efficacy

Quantitative data showed that the intervention affected the ICs self-awareness and –efficacy (Table 5). Over half of the ICs reported on being more aware of their own needs (*n*=15, 65%) and feeling more confident in handling their own feelings (*n*=15, 63%). ICs also indicated to feel more confident interacting with the ill person (*n*=11, 48%) and to be better at knowing (*n*=9, 38%) and assessing (*n*=11, 46%) their own capacities. Qualitative data revealed insights into how self-awareness and self-efficacy were improved. For example, the intervention had helped ICs reflecting upon finitude of life and their role as an IC, and thus to accept the situation and accompanying emotions. ICs reported being more aware of their own needs and the importance of self-care:

*„I learned (...) that I can work on myself (...). When I have a problem or fears I look at what does me good, (..) and that I do gymnastic exercises without having a bad conscience.“* (IC 18)

They felt recognized and valued in their unique burdens and needs as ICs:

*„It was definitely very useful and valuable for me to know (...) that there are people who think about us, the ICs. That not only patients are in such a difficult situation, but also we, the ICs.“* (IC 31)

Furthermore, ICs reported positive changes in dealing with the ill person by feeling more confident in communicating feelings:

*„What I remember best is dealing with my own fears, worries and needs, because that is what has burdened me most. (…) I gained more self-confidence in dialogue with my wife to talk about these things.”* (IC 28)

##### Potential burden

Despite the sensitive topics of the intervention, in quantitative data, most of the ICs (*n*=19, 83%) reported no further emotional distress due to participating. Sharing experiences with other persons in similar situations was described as very helpful (e.g. sense of common):

*„The feeling of not being alone (…), [to know] this happens to many people (…), that alone has helped me. (…) To release a little pressure (...) to see that other people are not doing well either when they see their loved ones will die.”* (IC 16)

##### Subjective burden of informal caregiving

Though most ICs reported subjective benefits, there was no significant change in the ICs burden as measured by the BSFC-s (*t*(21)=-1.65, *p*=.115, *N*=22; data not shown). At baseline, ICs on average showed a high burden of caregiving (*M*=16.4, *SD*=6.8, range 4-30). Three months later, the mean score dropped to a medium degree of burden (*M*=14.1, *SD*=7.4, range 4-29).

### Implementing the learned skills into daily life

#### Supporting factors

The most supporting factors (Table 7) for implementation to daily life were the ICs themselves and their motivation (*n*=23, 92%), other affected persons (*n*=12, 48%), information from TV, books or brochures (*n*=11, 46%), physicians or other healthcare professionals and the ill person (*n*=10, 42%). Thus, the ill person can be a hindering and supporting factor, which is also seen in the interviews. For example, one IC described improvements in her mother’s health as helpful for application of the newly acquired knowledge:

*„(…) my mother is getting fitter, which makes everything that I've learned a bit easier. Bit by bit, the overload (...) becomes less, which gives room for (...) for example self-care, making the implementation of the learned (...) easier.“* (IC 07)

In addition to the quantitative data, qualitative data revealed intervention-related factors to support implementation to daily life. Practical contents with specific tips and tutorials as well as additionally provided materials and recommendations were reported as particularly helpful, as one IC stated:

*„The contents were simply so practical, from my point of view it was immediately usable.“* (IC 34)

#### Hindering factors

At 3-months follow-up, internal and external factors were identified that hindered ICs implementing what they have learned into daily life (Table 8). Most ICs (*n*=15, 60%) stated “lack of time, stress*”* as a barrier, followed by their impaired own well-being (*n*=13, 54%), their job (*n*=13, 52%) and the ill person (*n*=10, 42%). These factors aligned with those reported in the interviews, with qualitative data illuminating the underlying mechanisms. For example, one IC described facing resistance as she wanted to talk to her partner about the power of attorney and the living will:

*„It couldn’t work out to start a conversation with my husband. (...) He always gives me the answer: I don't want to think about it.”* (IC 18)

**Table 8:** Hindering factors for practical implementation of the learned skills into daily life

*What made it difficult for you to implement what you have learned in the intervention?* *- Yes (multiple answers possible)*	***n/N* (%)**
Lack of time, stress	15/25 (60.0)
Impairment of my own well-being	13/24 (54.2)
Job/career	13/25 (52.0)
The ill person	10/24 (41.7)
My habits	10/25 (40.0)
My own health	7/24 (29.2)
Missing additional support services	4/24 (16.7)
Family, children, grandchildren	4/25 (16.0)
Lack of will or motivation	3/25 (12.0)
Financial situation	3/25 (12.0)
Friends	1/24 (4.2)

Moreover, ICs named their own habits and lack of motivation as hindering factors, both in quantitative (*n*=10, 40%; *n*=3, 12%) and qualitative data:

*„My own laziness, my own weaker self, and that I still don't have a living will although I was completely enthusiastic about it.“* (IC 02)

### Challenges of participating

Qualitative data revealed relevant challenges of participating in the intervention (Fig. 2). There were structural challenges like technical problems and lack of time, due to work, childcare or taking care of the ill person, as this quote illustrates:

*„ I worked in a full-time job, was in the hospital every day, and (...) took care of his cat. The time pressure (...) made it difficult for me.“* (IC 05)

Further, ICs described the group heterogeneity as demanding as they are facing different issues and questions due to various ages, relationships to the ill person and therefore different times in their life in general (e.g. newly married). Thereof, diverging needs and burdens could be derived. For instance, in one session most participants were older and looked back on a long-lasting relationship with the ill person. One younger IC reported facing different issues than the other participants, because of her specific stage of life:

*„We were faced with questions about starting a family and how to go on with life planning, and the older women, (…) the nature of accompaniment is different, after you've spent your whole life together.”* (IC 33)

Despite this challenge, the IC was still able to identify herself with the other participants in other aspects:

*“There were definitely individual comments from the other participants that made me think: yes, that's exactly how I've felt too.”* (IC 33)

Before starting the intervention, ICs recognized emotional challenges stemming from their uncertainty how they would deal with particular topics, from questioning their ability to seek and accept help, and from concerns about emotions possibly evoked by the intervention. As one IC narrated:

*„For me it was a challenge to go there, because all this is very emotional and I knew I would most likely cry a lot.“* (IC 11)

Despite such concerns, most of the ICs (n=19, 83%; Table 5) reported no further emotional distress after participating in the intervention.

### Potential for improvement

In the interviews, ICs were asked on how the intervention could be improved. While some ICs perceived the contents and the information density of the individual modules as sufficient, others requested adjustments, as illustrated by the following quotes:

*„What I missed a bit (...) [is the topic] how the ill person behaves and how I deal with it.“* (IC 18)

*„Sometimes [it was] too much content for too little time.”* (IC 07)

ICs heterogeneous needs also became apparent regarding the exchange among the participants. Some desired more opportunity for sharing experiences, for instance by closed group meetings and greater groups, as one participant stated:

*„Personally, I think it would be (...) good to have a closed group, to get to know each other and to learn more from the others, and thereby benefit.“* (IC 22)

At the same time, others appreciated the focus of the intervention on providing information and transferring of knowledge, as they explicitly did not seek for a support group.

Other named potentials for improvement were having more opportunities to ask questions and getting more practical tips, like checklists or exercises. Furthermore, ICs suggestions for improvement referred to the timeframe: different time of day, different day of the week or rhythm (e.g. every 2 weeks).

Regarding getting information about support services like this intervention, ICs wished to be informed by the professional caregivers:

*„I would have liked a nurse or a physician to talk to me personally about this [the intervention], so that you could feel (...)* individually addressed*.“* (IC 21)

Apart from the indications for possible improvements, other ICs stated to see no potential improvement:

*"I really don't know what could be improved."* (IC 11)

Moreover, ICs showed appreciation and gratefulness for the intervention and experienced the acquired knowledge as lasting:

*"What I find wonderful is that it is so sustainable. It is not as if you participated (...) and forgot about it, I still benefit from it. (...) It [the intervention] has really been like a gift for me."* (IC 32)

## Discussion

In this study, we evaluated a newly developed psychoeducational intervention named “Being an informal caregiver – strengthening resources”. The intervention aims to support and empower ICs of patients with incurable progressive cancer and non-cancer diseases by addressing relevant information-related, as well as physical, social and psychological needs. Main findings relate to the overall positive evaluation of the intervention with high satisfaction regarding its implementation, as well as various subjective benefits on the cognitive, emotional and behavioural level. The most apparent benefits were gaining knowledge, self-awareness, which can be seen in an increased focus on and awareness of their own needs as ICs, as well as an increased self-efficacy, as the ICs felt more confident in handling their situation. It was evident that the ICs felt seen and validated as a result of the intervention. Nevertheless, several factors were identified, which could support or hinder the implementation of the learned skills into daily life. Furthermore, motivations and challenges for participating were identified and opportunities for improving the intervention were discovered.

The ICs knowledge gain is reflected in the qualitative and quantitative data in many facets, for example in relation to care issues, disease progression, medication, legal aspects and support services, and therefore represents a major benefit of the intervention. This gain of knowledge in turn led to a feeling of preparedness and relief. It is well known that preparedness is not only needed with regard to practical aspects of caregiving, but also on the emotional level, since ICs have to cope with a broad range of emotions – from burden during caregiving to loss of the loved one [25]. To address these needs, we implemented the specific modules *own needs and emotions*, *self-care and own physical well-being* as well as *grief and loss*. ICs reported that the intervention had helped them reflecting on their role as an IC, the difficult situation they are confronted with as well as being more aware of their own needs and feeling more confident in dealing with the ill person. These benefits are reflected in changes on cognitive, emotional and on behavioural levels. Changes in the ICs everyday life, regarding themselves (e.g. increased self-care) as well as their interaction with the ill person (e.g. starting difficult conversations) can be found. Despite ICs difficulties of acknowledging and disclosing their own needs and accepting help [28], our psychoeducational intervention seems to succeed in its key goals. To increase the ICs preparedness, self-care and resilience can have a positive effect on ICs outcomes like caregiver burden [25–27, 63]. Psychoeducational, psychotherapeutic and mindfulness-based approaches might have a greater impact on ICs outcomes than other approaches [38]. However, further research is needed measuring the efficacy of our intervention on ICs outcomes, like psychological burden and quality of life.

Despite potentially burdensome topics of the intervention, ICs reported no additional emotional distress caused by intervention participation. On contrary, due to the positive experience, ICs felt more confident during the intervention. ICs had trust in information, considered the content clear and easy to understand, and difficult topics handled well. The fact that each module was provided by a palliative care professional experienced in this specific field as well as the professional, trustworthy environment in which the intervention was offered (university medical centre) might support this.

A major challenge of our study was the inclusion of ICs. In this case, the circumstances of the COVID-19 pandemic could be responsible, but low recruitment is a well-known problem in interventions supporting ICs and is reflected in numerous studies, also in the pre-pandemic era [24, 64]. In our study, most common barriers were lack of time and taking care of the ill person. These were also identified as hindering factors for implementing the learned skills into daily life. ICs feel highly responsible for the patients, put them in centre of care [65, 66] and feel uncomfortable leaving them even though they desire to take a break [28]. Our module-based intervention with short sessions and the opportunity to attend modules as required and possible could be a promising way to face this problem. Furthermore, web-based interventions might be useful for the overburdened population of ICs. Studies show that a web-based format of intervention is feasible [43], but comparisons between web-based and face-to-face interventions are missing. In our study, some ICs reported that they were only able to participate because the intervention was web-based, while others stated that they lacked personal contact and would prefer a face-to-face format. In both formats, ICs named potential challenges (web-based: e.g. technology, face-to-face: e.g. mobility and time), indicating a broad range of individual constraints, capabilities and needs among this heterogeneous group. Our study was not designed to answer this question and due to the small sample size, we are unable to provide any details or conclusion on the distinction between face-to-face and web-based participating. Future research on the acceptance of different intervention formats and their effects on ICs outcomes is required.

Furthermore, the group heterogeneity was described as challenging. In our study, ICs named the different ages and relationship to the ill person in the group as demanding, as it might have an impact on their individual needs and burdens. The heterogeneity is also shown in diverging expectations of participating, as some IC were specifically looking for exchange with other persons concerned, whereas others were seeking information and not sharing personal experiences. Due to the diversified group setting, heterogeneity is inevitable. For future implementations, this could be addressed at the beginning of a module, to emphasise the unifying aspect of being an IC and simultaneously the individuality of each participant. The high proportion of ICs of patients with a cancer disease might be an indication that ICs of non-cancer diseases did not feel addressed. When advertising the intervention, special attention should be paid to the interventions’ contents and aims, as well as to whom it is addressed.

From this study as well as other studies [24, 64] it is apparent, that there is a gap between ICs needs and their use of support services. Thus, further research is needed on overcoming this gap and improving participation. Our results show that IC wish to be informed about support services by their professional caregiver. For clinical practice this could mean that all professional caregivers - whether physicians, nurses or psychologists - should be aware of ICs needs, available support services and interventions, and actively offer them to their patients and ICs.

## Strengths and limitations

Our study has several strengths and limitations. One strength is the broad approach to ICs through the public recruitment strategy, the inclusion of ICs from both cancer and non-cancer patients and the possibility of early participation, not just when death is imminent or palliative care has already been implemented. Another advantage of our intervention is that it is offered in an open group setting, which allows for wider dissemination by reaching more ICs, in contrast to interventions that are designed as one-to-one contact by visit or telephone. Further, the intervention was manualised, which facilitates the implementation in other settings. By combining quantitative and qualitative methods to evaluate the intervention, we gained deeper insight into the ICs experiences of participating.

However, the generalizability of the results is limited due to the monocentric design of our study. Despite the broad recruitment approach, we only achieved a small sample size. We do not know which ICs did not participate to participate and for what reasons. It may also be that those ICs, who did participate, were in relatively good health and did not have too much caring work at that time. In addition, due to possible selection bias (mostly female, working and cancer patient ICs), our results may be less applicable to male ICs, those not working/retired and non-cancer patient ICs. Furthermore, there is no knowledge about the sustainability of the gained subjective benefits, as the evaluation ended with the 3-months follow-up. Transferability of the intervention might also be challenging, as the implementation requires experts from various professional groups, which might be difficult for smaller medical centres. However, the experts for the intervention do not have to be part of the same team or organization, which may be an advantage in terms of expert recruitment. In order to reduce resource requirements, the intervention could be offered three to four times a year, for example, instead of a continuous weekly program.

## Conclusion

Strengthening ICs is important because they feel unprepared and the tasks and role as an IC entail a variety of burdens. This mixed methods pilot study shows an overall positive evaluation of the intervention “Being an informal caregiver – strengthening resources”, in terms of both its content and its implementation. Important goals pursued by a psychoeducational intervention, like improvement of knowledge, self-awareness and self-efficacy, were achieved, with positive changes reported at cognitive, emotional and behavioural levels. This manualised intervention has the potential to have a positive impact on ICs. A multicentre randomized prospective study is planned to measure the efficacy of the intervention on ICs outcomes.

Nevertheless, it was a major challenge to include ICs in our intervention, which is a common problem in interventions supporting ICs of patients with progressive incurable diseases. It is particularly important to develop strategies to effectively disseminate available support services and increase participation, including that of non-cancer ICs. In our study, ICs emphasized the key role of professional caregivers in informing about support services. Therefore, professionals have to be equipped with relevant information about support services and about when to refer ICs to such services, also in non-cancer settings. Furthermore, new intervention formats, such as web-based formats, might lower the threshold for participation in this burdened population. Future research of our study group aims to examine the acceptance of different intervention formats and their effects on participation.

### Supplementary Information

**Supplementary Material 1.** 

**Supplementary material 2.** 

**Supplementary material 3.** 

**Supplementary material 4.** 

**Supplementary material 5.** 

**Supplementary material 6.** 

## Data Availability

The datasets generated and/or analysed during the current study are not publicly available due to reasons of privacy but are available from the corresponding author (TT) on reasonable request.

## Abbreviations

IC: Informal caregiver
COVID-19: Coronavirus Disease 2019
LAC: Last Aid Course
KoMPaC: Curriculum for developing training programs in palliative care
TIDieR: Template for Intervention Description and Replication Checklist
heiQ-program: Health Education Impact Questionnaire program scale
BSFC-s: Burden Scale for Family Caregivers
M: mean
SD: standard deviation
GRAMMS: Good Reporting of A Mixed Methods Study Checklist
