# Correction: “Being an informal caregiver – strengthening resources”: mixed methods evaluation of a psychoeducational intervention supporting informal caregivers in palliative care

**DOI:** 10.1186/s12904-024-01444-0

**Published:** 2024-05-02

**Authors:** Tabea Theißen, Anneke Ullrich, Karin Oechsle, Julia Wikert, Carsten Bokemeyer, Aneta Schieferdecker

**Affiliations:** 1 Palliative Care Unit, Department of Oncology, Haematology and BMT, University Medical Centre Hamburg-Eppendorf, Martinistr. 52, 20246 Hamburg, Germany; 2 Department of Palliative Medicine, LMU University Hospital, Munich, Germany

**Correction: BMC Palliat Care 23, 95 (2024).**

10.1186/s12904-024-01428-0

Following publication of the original article [1], the author reported that they missed to include a funder.

The Funding section should be updated from:

Open Access funding enabled and organized by Projekt DEAL. This study received funding by the “Förderverein der Palliativversorgung am UKE e.V” within the Full Professorship for Palliative Care with Focus on Informal Caregivers Research. The funders of the study had no role in the study design, data collection and analysis, decision to publish, or preparation of the manuscript.

To

Open Access funding enabled and organized by Projekt DEAL. This study received funding by the “Förderverein der Palliativversorgung am UKE e.V” within the Full Professorship for Palliative Care with Focus on Informal Caregivers Research. **We acknowledge financial support from the Open Access Publication Fund of UKE - Universitätsklinikum Hamburg-Eppendorf.** The funders of the study had no role in the study design, data collection and analysis, decision to publish, or preparation of the manuscript.

Moreover, the Supplementary materials were updated to a clearer version.

The original article has been updated.
