# Supplementary material for: “Being an informal caregiver – strengthening resources”: mixed methods evaluation of a psychoeducational intervention supporting informal caregivers in palliative care
Source: BMC Palliat Care. 2024 Apr 11;23:95. doi: 10.1186/s12904-024-01428-0 (PMC11007958; doi:10.1186/s12904-024-01428-0)
Supplement: Supplementary file 2 — Supplementary material 2. [file 12904_2024_1428_MOESM2_ESM.docx]

**Suppl. File 2.** Semi-structured interview guide

| Date: | I__I__I__I__I__I__I__I__I |
| --- | --- |
| Gender: | m / w / d |
| Age: | _______ |

**“Being an informal caregiver – strengthening resources”
Interview guide: Evaluation of an intervention supporting informal caregivers of patients with progressive incurable diseases.**

Thank you for your participation! The interview will last approx. 45-60 min. and is part of the evaluation of the intervention for informal caregivers (ICs). The aim of this interview is to learn more about your subjective experience with the intervention: benefits of participating in the intervention, the implementation and content, as well as barriers of participation and practical implementation to daily life.

If you agree, we would like to record the interview. The audio recording will be deleted after the written transcription. Audio recording agreed □ YES □ NO

**ICs information**

Beginning, would you tell me if your relative, because of whom you participated in the intervention, is currently still alive? □ alive □ deceased

What is/was your relationship with the ill person with?

- Life partner or spouse
- Son/daughter
- Father/Mother
- Other: _____________________________________

Which disease does/did the ill person have?

- Oncological disease
- Heart-lung disease
- Multimorbid disease
- Other: _____________________________________

Do/did you live in the same household as the ill person?

- Yes
- No

Does/did the ill person have a care degree? (Additional support from outpatient care service)

- Yes
- No

In which format did you attend the intervention?

- web-based
- face-to-face

How many modules did you attend?

- At least 5
- Less than 5: _________________________________

**Before the intervention**

1. How did you become aware of the intervention?

- Have you seen flyers or posters of the intervention? If so, where?
- What aroused your interest? Why did you decide to participate in the intervention?

1. What were your expectations before participating?

- What did you hope to get out of the intervention?

**Positive/negative aspects of the intervention**

1. How did you feel during the individual modules?

- How did you feel about the group situation (exchange with others, lecturers)?
- Were there any topics/questions that touched you? How did you feel?

1. For you personally, what were positive aspects of the intervention?
2. What did you personally not (so much) like about the intervention?

- Where do you need more information or input?
- Which situation would you most likely rate as negative?

**Barriers/challenges for participating**

1. What do you think were the biggest challenges/difficulties in the intervention and why?

- What were barriers for you personally to participate in each module?
- Was there anything that made it difficult for you to get involved?

*If you did not participate in individual models:*

- Were there any particular reasons why you did not participate in the modules?

**Practical implementation to daily life and subjective benefits of the intervention**

1. Have there been any changes in your everyday life/in dealing with the ill person as a result of the intervention?

- How did you notice that the intervention helped/did not help you?
- Were there any changes in your feelings or attitudes towards the intervention during the series of modules?
- Were there any unexpected effects/changes for you? - both negative and positive

1. What did you take away from the intervention for yourself (as an IC)?

*Follow-up if not already mentioned:*

- What was the biggest benefit for you?

*Follow-up questions if no:*

- What would have had to be different for you to have taken something away?

1. What made it easy for you to implement what you had learned?
2. What made it difficult for you?

- Was there anything about your thoughts or behaviour that made it difficult for you?
- Did you find it difficult to apply what you had learned in everyday life for emotional reasons?

1. Would you recommend the intervention to others?

- Yes
- No

**Suggestions and wishes regarding the implementation and further topics/contents**

1. Have there been parts in the intervention that were not so important in your case, but could be helpful for other ICs?
2. In our experience, there is always room for improvement. What tips would you give to the people who developed and delivered this intervention to make it as helpful and meaningful as possible? (To better meet the needs of ICs)

- Changes regarding the organisation/structure of the intervention?
- Regarding the topics/modules?
- Regarding the accessibility of ICs?

**Closing/room for questions**

1. Is there anything else that would be important to you personally that we haven't talked about yet?

Thank you for your participation, we wish you all the best for the future!
