# Supplementary material for: “Being an informal caregiver – strengthening resources”: mixed methods evaluation of a psychoeducational intervention supporting informal caregivers in palliative care
Source: BMC Palliat Care. 2024 Apr 11;23:95. doi: 10.1186/s12904-024-01428-0 (PMC11007958; doi:10.1186/s12904-024-01428-0)
Supplement: Supplementary file 3 — Supplementary material 3. [file 12904_2024_1428_MOESM3_ESM.pdf]

Suppl. File 3. Good Reporting of a Mixed Methods Study (GRAMMS) Checklist

Guideline	Section: Page
(1) Describe the justification for using a mixed methods approach to the research question	Methods: p. 8
(2) Describe the design in terms of the purpose, priority and sequence of methods	Methods: pp.7-8
(3) Describe each method in terms of sampling, data collection and analysis	Methods: pp. 7-11
(4) Describe where integration has occurred, how it has occurred and who has participated in it	Methods: p. 11
(5) Describe any limitation of one method associated with the present of the other method	Discussion: pp. 25-26
(6) Describe any insights gained from mixing or integrating methods	Discussion: pp. 23-25

O'Cathain A, Murphy E, Nicholl J. The quality of mixed methods studies in health services research. J Health Serv Res Policy. 2008;13: 92-98.
