# Supplementary material for: “Being an informal caregiver – strengthening resources”: mixed methods evaluation of a psychoeducational intervention supporting informal caregivers in palliative care
Source: BMC Palliat Care. 2024 Apr 11;23:95. doi: 10.1186/s12904-024-01428-0 (PMC11007958; doi:10.1186/s12904-024-01428-0)
Supplement: Supplementary file 4 — Supplementary material 4. [file 12904_2024_1428_MOESM4_ESM.pdf]

Suppl. File 4: Category system of the qualitative evaluation, including explanation of categories and illustrative quotes.

Category	Subcategory	Explanation of category	Illustrative quote
Intention of participating		Interest in / expectations of the intervention	
	ICs in focus	Explicit focus on ICs and their needs, including the topic self-care	*„[My interest was aroused] because there, the informal caregiver were placed in the foreground."* (IC 22)
	Information / topics / contents	Specific topics and contents; receiving information, practical tips and / or addresses of support services	*„Information, gaining knowledge (...) I just wanted to know: what to expect and who to ask if I don't know what to do."* (IC 24)
	Contact / exchange (with other ICs)	Desire or expectation to get in contact / exchange with other ICs, feeling of not being alone	*„My expectation [was] (...) to experience relief through the exchange with other informal caregivers."* (IC 22)
	Dealing with difficult topics	Dealing with potentially burdensome topics, e.g. grief	*„Grieving, to deal with the topic (...), can it affect me or not and how do I deal with it."* (IC 31)
	Strengthen own resources	Strengthening of own resources, e.g. dealing with own emotions and fears, topic self-care	*„I expected (...) to get (...) information on how I can cope better with the situation, (...) how I can create resources for myself."* (IC 28)
	Self-efficacy	Desire for self-efficacy, "to become active", "to be able to do something"	*„My interest was (...) what can I do (...), [because] I want to (...) actively support my loved one."* (IC 04)
	Better way of dealing with the ill person	Acquiring a better understanding of and / or better dealing with the	*„Expectation (...) that I would get (...) more information and in the best case I could (...) deal with my dad differently then."* (IC11)
Challenges of participating		Structural and / or emotional barriers to the participation in and / or during the intervention	
	Technical problems	Technical problems due to which participation in the intervention (or parts of) was not possible	*„At one point [was the internet connection] was so unstable, when I was kicked out the second time and the third time, I said to myself, then it shouldn't be."* (IC 21)
	Time	Lack of time to attend the intervention, e.g. due to work, care of the ill person, child care	*„This time problem. I worked in a full-time job, was in the hospital every day, and (...) took care of his cat. The time pressure (...) made it difficult for me."* (IC 05)
	Group heterogeneity	Heterogeneity of participants, e.g. in terms of age or relationship to the ill person (spouse, child, etc)	*„We were faced with questions about starting a family and how to go on with life planning, and the older women, (...) the nature of accompaniment is different, after you've spent your whole life together."* (IC 33)#
	Being able to allow topic	Challenge to be able to allow or to deal with a particular topic	*„Dealing with the finitude of life (...) and discussions about living wills, health care proxy and wills (...) is emotionally difficult."* (IC 21)
	Being able to allow help	Challenge to acknowledge that you yourself needing help, seeking help and / or accepting help	*„[The challenge is] to start doing something (...), this `I'm looking for something now because I urgently need (...) help here`."* (IC 07)
	Fear of emotions	Fear of emotions, experiencing deep feelings, crying, feeling ashamed	*„For me it was a challenge to go there, because all this is very emotional and I knew I would most likely cry a lot."* (IC 11)

Category	Subcategory	Explanation of category	Illustrative quote
Subjective benefits		Benefits of the intervention, which can be on an emotional, cognitive and / or behavioural level; in some cases, these have led to concrete actions and changes in everyday life	<u>cognitive level</u>: *„Due to the intervention, I realised that I should take care of myself more often.“* (IC 04)
			<u>emotional level</u>: *„(...) to gain the feeling that it is okay to feel a certain way and also to accept oneself with one's own fears and worries.“* (IC 33)
			<u>behavioural level</u>: *„We immediately looked for a local palliative care physician, here where we live (...) we go there every four weeks.“* (IC 24)
	Being "seen" as an IC	To be seen as IC in its needs as well as the existing burdens and *dual role*[a]	*„It was definitely very useful and valuable for me to know (...) that there are people who think about us, the ICs. That not only patients are in such a difficult situation, but also we, the ICs.“* (IC 31)
	Experience of not being alone	Relief from the experience of not being alone, that others are also affected and feel the same way	*„The feeling of not being alone (...), [to know] this happens to many people (...), that alone has helped me.“* (IC 16)
	Acceptance (situation, emotions, support)	Acceptance of the situation or accompanying feelings and / or acceptance of help and support services	*„[I picked up] talking about it, about the situation and that if anything ever happens again, I'll get help and support much earlier.“* (IC 02)
	Help through information	Help through information, practical tips, provided materials, learning from other participants	*„I learned (...) who I can contact and also how it works when he dies, who will take care of him and that the funeral home will do a lot. That was a real benefit for me.“* (IC 05)
	Understanding palliative care	Awareness of the goals, principles and options of palliative care, including timely integration of these	*„I realized (...) that palliative care does not just start when the end of life is very near, but even before that (...) that was a very important realisation for me.“* (IC 28)
	Starting point for conversations	Starting point for conversations with ill person and / or other ICs, e.g. about health care proxy, patients living will, fears and death	*„Yes, of course there were changes. For the first time I discussed some issues with my sister.“* (IC 03)
	Self-care	Taking care of oneself, being aware of one's own needs, taking time outs	*„I have (...) learned, quite clearly, I have to take care of myself, which I didn't do before.“* (IC 05)
	Self-reflection	Dealing with the situation and oneself, e.g. being aware of the difficult situation, the finitude of life, one's *dual role** as IC	*„(...) because at that moment I was able to think about my current situation myself and also to realise again what situation I am actually in and to become aware that it was difficult.“* (IC 22)
	Changes in dealing with the ill person	Changes in dealing with ill person, e.g. through greater confidence in communication, in dealing with specific issues such as eating and drinking	*„What I remember best is dealing with my own fears, worries and needs, because that is what has burdened me most. (...) I gained more self-confidence in dialogue with my wife to talk about these things.“* (IC 28)
	Security	Security through confirmation of prior knowledge and experience, through the information ''you cannot do anything wrong'', through participation during the course of intervention	*„That helped me a lot, (...) to know that I can't do anything wrong.“* (IC 32)
			„ At the beginning I was skeptical, maybe a little bit afraid, because of the emotional issues, but in the course it was okay, I felt really well taken care of.“ (IC11)

Category	Subcategory	Explanation of category	Illustrative quote
Implementing the learned skills into daily life		Supporting and hindering factors for the implementation of learned content	
	Supporting factors	Factors that facilitated the implementation of the learned, e.g. materials provided, practical tips, practical content	*„(...) my mother is getting fitter, which makes everything that I've learned a bit easier. Bit by bit, the overload (...) becomes less, which gives room for (...) for example self-care, making the implementation of the learned (...) easier.“* (IC 07)<br>*„The contents were simply so practical, from my point of view it was immediately usable.“* (IC 34)
	Hindering factors	Factors that made it difficult to implement what has been learned, e.g. "own laziness", ”own weaker self", psychological tension, lack of time, the ill person	*„My own laziness, my own weaker self, and that I still don't have a living will although I was completely enthusiastic about it.“* (IC 02)<br>*„It couldn’t work out to start a conversation with my husband. (...) He always gives me the answer: I don't want to think about it.”* (IC 18)
Potential for improvement		Mentioned possibilities for improvement on the content or structural level	
	Topics and contents	Criticism of specific topics / content or naming of additional topics / content	*„What I missed a bit (...) [is the topic] how the ill person behaves and how I deal with it.“* (IC 18)
	Information density	Desire for different ratio of information to time (within a module)	*„Sometimes [it was] too much content for too little time.”* (IC 07)
	Opportunity to ask questions	More time for questions / addressing the individual situation	*„The information were very specific and my expectation was (...) to have a rough overview (...) and then (...) to ask individual questions.“* (IC 22)
	Practical tips, checklists, exercises	Desire for practical exercises as part of the intervention, exercises to do at home, practical tips, checklists	*„(...) what I have to do if my loved one dies, (...) from one day to the next. (...) I would like to have a kind of (...) checklist.“* (IC 04)
	Exchange among the participants	More exchange among participants (e.g. through introduction rounds, fixed group of participants, more participants, encourage more exchange) or more demarcation from self-help group, preliminary talk before participation	*„Personally, I think it would be (...) good to have a closed group, to get to know each other and to learn more from the others, and thereby benefit.“* (IC 22)
	Information materials	Desire for listing of points of contact (including those in neighboring regions / states), handouts for training, and literature recommendations	*„Contact points that offer consulting or accompaniment with psycho-oncological or psychotherapeutic support, which consulting services that exist in Hamburg and in the neighbouring states.“* (IC 31)
	Date	Suggestions on date, e.g. different time, different day of the week, different rhythm (every 2 weeks)	*„As an improvement I would (...) say that the support intervention is offered during the week but also at the weekend, (...) where people who are working might have a little bit more time.”* (IC 19)
	Accessibility of ICs	Suggestions / ideas for reaching ICs, flyer design	*„I would have liked a nurse or a physician to talk to me personally about this [the intervention], so that you could feel (...) individually addressed.“* (IC 21)
