# Supplementary material for: “Being an informal caregiver – strengthening resources”: mixed methods evaluation of a psychoeducational intervention supporting informal caregivers in palliative care
Source: BMC Palliat Care. 2024 Apr 11;23:95. doi: 10.1186/s12904-024-01428-0 (PMC11007958; doi:10.1186/s12904-024-01428-0)
Supplement: Supplementary file 5 — Supplementary material 5. [file 12904_2024_1428_MOESM5_ESM.pdf]

Suppl. File 5. Satisfaction with the implementation of the intervention after each module

**Module 1 (N=17)**<br>**Hands-on care: tips and strategies for providing care at home**			
**Items**	***M* (*SD*)**	**Range[a]**	**Agree[b]** ***n* (%)**
**HeiQ-program**			
1. I will tell people that the module is very worthwhile	4.5 (0.6)	3-5	16 (94.1)
2. The module has helped me set reasonable and achievable goals	3.8 (0.7)	2-5	13 (76.5)
3. I trust the information and advice given in the module	4.5 (0.5)	4-5	17 (100.0)
4. The module was very well organized.	4.5 (0.5)	4-5	17 (100.0)
5. Taking part in the module was worth my time and effort	4.1 (0.8)	2-5	15 (88.2)
6. Difficult topics and discussions were handled well	4.4 (0.6)	3-5	16 (94.1)
7. The module content was very relevant to me and my situation	3.9 (1.1)	1-5	12 (70.6)
8. Everyone had the chance to speak if they wanted to	4.8 (0.4)	4-5	17 (100.0)
9. The group worked very well together	4.3 (1.0)	1-5	15 (93.8)
**Additional items**			
10. My expectations of the module were met[c]	4.2 (0.6)	3-5	15 (88.2)
11. The subject matter provided was clear and easy to understand[c]	4.7 (0.5)	4-5	17 (100.0)
**Module 2 (N=21)**<br>**Getting prepared: Information about social and legal issues**			
**Items**	***M* (*SD*)**	**Range[a]**	**Agree[b]** ***n* (%)**
**HeiQ-program**			
1. I will tell people that the module is very worthwhile	4.5 (1.1)	1-5	19 (90.5)
2. The module has helped me set reasonable and achievable goals	3.9 (1.1)	1-5	16 (76.2)
3. I trust the information and advice given in the module	4.5 (0.8)	2-5	19 (90.5)
4. The module was very well organized.	4.2 (0.9)	2-5	16 (76.2)
5. Taking part in the module was worth my time and effort	4.4 (1.0)	2-5	18 (85.7)
6. Difficult topics and discussions were handled well	4.4 (0.9)	2-5	18 (85.7)
7. The module content was very relevant to me and my situation	4.3 (1.0)	2-5	16 (76.2)
8. Everyone had the chance to speak if they wanted to	4.3 (0.9)	2-5	17 (81.0)
9. The group worked very well together	3.7 (1.2)	1-5	11 (57.9)
**Additional items**			
10. My expectations of the module were met[c]	4.3 (1.1)	2-5	17 (81.0)
11. The subject matter provided was clear and easy to understand[c]	4.7 (7.3)	2-5	20 (95.2)
**Module 3 (N=19)**<br>**Questions, uncertainties and concerns about grief and loss**			
**Items**	***M* (*SD*)**	**Range[a]**	**Agree[b]** ***n* (%)**
**HeiQ-program**			
1. I will tell people that the module is very worthwhile	4.0 (0.9)	2-5	15 (78.9)
2. The module has helped me set reasonable and achievable goals	3.6 (0.9)	2-5	10 (52.6)
3. I trust the information and advice given in the module	4.4 (0.7)	3-5	17 (89.5)
4. The module was very well organized.	4.2 (0.9)	2-5	16 (84.2)
5. Taking part in the module was worth my time and effort	4.0 (1.1)	1-5	15 (78.9)
6. Difficult topics and discussions were handled well	4.3 (1.0)	2-5	14 (73.7)
7. The module content was very relevant to me and my situation	4.1 (0.8)	3-5	14 (73.7)
8. Everyone had the chance to speak if they wanted to	4.7 (0.5)	4-5	18 (100.0)
9. The group worked very well together	4.3 (1.3)	0-5	15 (83.3)
**Additional items**			
10. My expectations of the module were met[c]	4.0 (1.0)	2-5	14 (73.7)
11. The subject matter provided was clear and easy to understand[c]	4.4 (0.8)	3-5	16 (84.2)
**Module 4 (*N*=18)**<br>**Strategies to cope with own needs and emotions**			
**Items**	***M* (*SD*)**	**Range[a]**	**Agree[b]** ***n* (%)**

Items	*M* (*SD*)	Range[a]	Agree[b] *n* (%)
**HeiQ-program**			
1. I will tell people that the module is very worthwhile	4.6 (0.6)	3-5	17 (94.4)
2. The module has helped me set reasonable and achievable goals	3.8 (0.6)	3-5	13 (72.2)
3. I trust the information and advice given in the module	4.6 (0.6)	3-5	17 (94.4)
4. The module was very well organized.	4.5 (0.7)	3-5	16 (88.9)
5. Taking part in the module was worth my time and effort	4.4 (0.7)	3-5	16 (88.9)
6. Difficult topics and discussions were handled well	4.5 (0.6)	3-5	17 (94.4)
7. The module content was very relevant to me and my situation	4.2 (0.8)	3-5	14 (77.8)
8. Everyone had the chance to speak if they wanted to	4.4 (0.9)	3-5	14 (77.8)
9. The group worked very well together	3.9 (1.2)	1-5	13 (72.2)
**Additional items**			
10. My expectations of the module were met[c]	4.1 (0.9)	2-5	14 (77.8)
11. The subject matter provided was clear and easy to understand[c]	4.6 (0.6)	3-5	17 (94.4)
**Module 5 (*N*=21)** **Strategies for handling changes in the disease progression**			
**Items**	***M* (*SD*)**	**Range[a]**	**Agree[b] *n* (%)**
**HeiQ-program**			
1. I will tell people that the module is very worthwhile	4.4 (0.7)	3-5	19 (90.5)
2. The module has helped me set reasonable and achievable goals	3.1 (1.4)	0-5	12 (57.1)
3. I trust the information and advice given in the module	4.8 (0.4)	4-5	21 (100.0)
4. The module was very well organized.	4.6 (0.7)	3-5	19 (90.5)
5. Taking part in the module was worth my time and effort	4.1 (1.2)	1-5	16 (76.2)
6. Difficult topics and discussions were handled well	4.5 (0.7)	3-5	19 (90.5)
7. The module content was very relevant to me and my situation	3.8 (1.5)	0-5	15 (71.4)
8. Everyone had the chance to speak if they wanted to	4.9 (0.4)	4-5	21 (100.0)
9. The group worked very well together	3.5 (1.4)	0-5	11 (52.4)
**Additional items**			
10. My expectations of the module were met[c]	3.8 (1.2)	1-5	15 (71.4)
11. The subject matter provided was clear and easy to understand[c]	4.9 (0.4)	4-5	21 (100.0)
**Module 6 (*N*=17)** **Practical exercises for self-care and own physical well-being**			
**Items**	***M* (*SD*)**	**Range[a]**	**Agree[b] *n* (%)**
**HeiQ-program**			
1. I will tell people that the module is very worthwhile	4.1 (0.9)	3-5	12 (70.6)
2. The module has helped me set reasonable and achievable goals	3.7 (1.1)	2-5	10 (58.8)
3. I trust the information and advice given in the module	4.5 (0.7)	3-5	15 (88.2)
4. The module was very well organized.	4.0 (0.9)	2-5	12 (70.6)
5. Taking part in the module was worth my time and effort	3.8 (1.0)	2-5	8 (47.1)
6. Difficult topics and discussions were handled well	4.1 (0.6)	3-5	14 (87.5)
7. The module content was very relevant to me and my situation	3.5 (1.5)	1-5	6 (37.5)
8. Everyone had the chance to speak if they wanted to	4.8 (0.5)	3-5	15 (93.8)
9. The group worked very well together	4.5 (0.6)	3-5	15 (93.8)
**Additional items**			
10. My expectations of the module were met[c]	3.6 (1.3)	1-5	9 (56.3)
11. The subject matter provided was clear and easy to understand[c]	4.5 (0.8)	3-5	13 (81.3)

Abbreviations: *M*, Mean; *SD*, Standard deviation; [a] range on a Likert response scale from (0 *strongly disagree* - 5 *strongly agree*) [b]versus (*strongly) disagree/disagree somewhat/agree somewhat* [c]Study specific items not included in the original heiQ-program
