# Supplementary material for: “Being an informal caregiver – strengthening resources”: mixed methods evaluation of a psychoeducational intervention supporting informal caregivers in palliative care
Source: BMC Palliat Care. 2024 Apr 11;23:95. doi: 10.1186/s12904-024-01428-0 (PMC11007958; doi:10.1186/s12904-024-01428-0)
Supplement: Supplementary file 6 — Supplementary material 6. [file 12904_2024_1428_MOESM6_ESM.pdf]

Suppl. File 6. ICs benefits of participating after each module

**Module 1 (N=17)**<br>**Hands-on care: tips and strategies for providing care at home**				
		***M* (*SD*)**	**Range[a]**	**Agree[b]**
**Topic**	*Comparing my current situation to my situation before the intervention, I now...*			***n* (%)**
**Gaining knowledge**	know better where to find help and support as a ICs	3.9 (0.7)	2-5	14 (82.4)
	have more answers to important questions	3.9 (0.9)	1-5	14 (82.4)
**Self-awareness and -efficacy**	am more aware of my own needs as a ICs	3.8 (0.9)	2-5	13 (76.5)
	feel more confident handling feelings regarding this topics	3.9 (0.8)	2-5	13 (76.5)
	feel more confident interacting with the ill person	3.8 (1.1)	1-5	12 (70.6)
	am better at estimating, what I am capable of	3.5 (1.2)	1-5	11 (64.7)
	know my own abilities better now	3.5 (1.0)	1-5	9 (52.9)
**Potential burden**	do NOT feel more anxious, worried or depressed	4.1 (1.1)	1-5	15 (88.2)

**Module 2 (N=21)**<br>**Getting prepared: Information about social and legal issues**				
		***M* (*SD*)**	**Range[a]**	**Agree[b]**
**Topic**	*Comparing my current situation to my situation before the intervention, I now...*			***n* (%)**
**Gaining knowledge**	know better where to find help and support as a ICs	4.1 (0.9)	2-5	17 (81.0)
	have more answers to important questions	2.9 (1.3)	0-5	16 (76.2)
**Self-awareness and -efficacy**	am more aware of my own needs as a ICs	3.5 (1.0)	2-5	11 (52.4)
	feel more confident handling feelings regarding this topics	3.2 (0.8)	2-5	8 (38.1)
	feel more confident interacting with the ill person	3.2 (1.0)	2-5	9 (42.9)
	am better at estimating, what I am capable of	3.5 (1.1)	1-5	12 (57.1)
	know my own abilities better now	3.2 (1.2)	0-5	11 (52.4)
**Potential burden**	do NOT feel more anxious, worried or depressed	4.0 (0.8)	3-5	14 (66.7)

**Module 3 (N=19)**<br>**Questions, uncertainties and concerns about grief and loss**				
		***M* (*SD*)**	**Range[a]**	**Agree[b]**
**Topic**	*Comparing my current situation to my situation before the intervention, I now...*			***n/N* (%)**
**Gaining knowledge**	know better where to find help and support as a ICs	3.7 (1.1)	1-5	11/19 (57.9)
	have more answers to important questions	3.4 (1.1)	1-5	11/19 (57.9)
**Self-awareness and -efficacy**	am more aware of my own needs as a ICs	3.5 (1.1)	1-5	10/19 (52.6)
	feel more confident handling feelings regarding this topics	3.2 (1.1)	1-5	8/19 (42.1)
	feel more confident interacting with the ill person	3.2 (1.2)	1-5	8/18 (44.4)
	am better at estimating, what I am capable of	3.1 (1.1)	1-5	6/19 (31.6)
	know my own abilities better now	2.8 (1.1)	1-5	4/19 (21.1)
**Potential burden**	do NOT feel more anxious, worried or depressed	4.0 (0.7)	3-5	15/19 (78.9)

**Module 4 (*N*=18)**<br>**Strategies to cope with own needs and emotions**				
		***M* (*SD*)**	**Range[a]**	**Agree[b]**
**Topic**	*Comparing my current situation to my situation before the intervention, I now...*			***n* (%)**
**Gaining knowledge**	know better where to find help and support as a ICs	4.2 (0.8)	2-5	16 (88.9)
	have more answers to important questions	3.9 (1.1)	2-5	12 (66.7)
**Self-awareness**	am more aware of my own needs as a ICs	4.2 (1.0)	2-5	14 (77.8)

**and -efficacy**	feel more confident handling feelings regarding this topics	3.8 (1.0)	2-5	13 (72.2)
	feel more confident interacting with the ill person	3.6 (0.9)	2-5	11 (61.1)
	am better at estimating, what I am capable of	3.6 (1.0)	2-5	12 (66.7)
	know my own abilities better now	3.5 (0.9)	2-5	10 (55.6)
**Potential burden**	do NOT feel more anxious, worried or depressed	4.1 (0.9)	2-5	14 (77.8)

Module 5 (*N*=21)
Strategies for handling changes in the disease progression

		***M* (*SD*)**	**Range[a]**	**Agree[b]**
**Topic**	*Comparing my current situation to my situation before the intervention, I now...*			***n* (%)**
**Gaining knowledge**	know better where to find help and support as a ICs	3.3 (1.4)	1-5	10 (47.6)
	have more answers to important questions	3.8 (1.1)	1-5	15 (71.4)
**Self-awareness**	am more aware of my own needs as a ICs	2.7 (1.5)	0-5	9 (42.9)
**and -efficacy**	feel more confident handling feelings regarding this topics	3.3 (1.4)	0-5	13 (61.9)
	feel more confident interacting with the ill person	3.3 (1.2)	0-5	11 (52.4)
	am better at estimating, what I am capable of	2.9 (1.4)	0-5	8 (38.1)
	know my own abilities better now	2.7 (1.5)	0-5	7 (33.3)
**Potential burden**	do NOT feel more anxious, worried or depressed	4.2 (0.7)	3-5	18 (85.7)

Module 6 (*N*=17)
Practical exercises for self-care and own physical well-being

		***M* (*SD*)**	**Range[a]**	**Agree[b]**
**Topic**	*Comparing my current situation to my situation before the intervention, I now...*			***n/N* (%)**
**Gaining knowledge**	know better where to find help and support as a ICs	2.9 (1.1)	1-5	4/16 (25.0)
	have more answers to important questions	3.2 (1.0)	2-5	5/16 (31.3)
**Self-awareness**	am more aware of my own needs as a ICs	3.6 (0.9)	2-5	7/16 (43.8)
**and -efficacy**	feel more confident handling feelings regarding this topics	2.9 (0.9)	1-5	2/16 (12.5)
	feel more confident interacting with the ill person	2.9 (0.9)	2-5	3/16 (18.8)
	am better at estimating, what I am capable of	2.9 (1.0)	1-5	3/16 (18.8)
	know my own abilities better now	3.1 (0.8)	2-5	4/16 (25.0)
**Potential burden**	do NOT feel more anxious, worried or depressed	4.5 (0.7)	3-5	14/16 (87.5)

Abbreviations: *M*, Mean; *SD*, Standard deviation; ICs, informal caregivers[a] range on a 6-point response scale from (0 *strongly disagree* - 5 *strongly agree*) [b]versus (*strongly) disagree/disagree somewhat/agree somewhat*
